# Oncostatin M receptor β deficiency attenuates atherogenesis by inhibiting JAK2/STAT3 signaling in macrophages

**DOI:** 10.1194/jlr.M074112

**Published:** 2017-04-28

**Authors:** Xin Zhang, Jing Li, Juan-Juan Qin, Wen-Lin Cheng, Xueyong Zhu, Fu-Han Gong, Zhigang She, Zan Huang, Hao Xia, Hongliang Li

**Affiliations:** Department of Cardiology, Renmin Hospital,* Wuhan University, Wuhan, China; Institute of Model Animals,† Wuhan University, Wuhan, China; Medical Research Institute, School of Medicine,§ Wuhan University, Wuhan, China; College of Life Science,** Wuhan University, Wuhan, China

**Keywords:** atherosclerosis, inflammation, Janus kinase 2/signal transducer and activator of transcription 3

## Abstract

Oncostatin M (OSM) is a secreted cytokine mainly involved in chronic inflammatory and cardiovascular diseases through binding to OSM receptor β (OSMR-β). Recent studies demonstrated that the presence of OSM contributed to the destabilization of atherosclerotic plaque. To investigate whether OSMR-β deficiency affects atherosclerosis, male OSMR-β^−/−^ApoE^−/−^ mice were generated and utilized. Here we observed that OSMR-β expression was remarkably upregulated in both human and mouse atherosclerotic lesions, which were mainly located in macrophages. We found that OSMR-β deficiency significantly ameliorated atherosclerotic burden in aorta and aortic root relative to ApoE-deficient littermates and enhanced the stability of atherosclerotic plaques by increasing collagen and smooth muscle cell content, while decreasing macrophage infiltration and lipid accumulation. Moreover, bone marrow transplantation of OSMR-β^−/−^ hematopoietic cells to atherosclerosis-prone mice displayed a consistent phenotype. Additionally, we observed a relatively reduced level of JAK2 and signal transducer and activator of transcription (STAT)3 in vivo and under Ox-LDL stimulation in vitro. Our findings suggest that OSMR-β deficiency in macrophages improved high-fat diet-induced atherogenesis and plaque vulnerability. Mech­anistically, the protective effect of OSMR-β deficiency on atherosclerosis may be partially attributed to the inhibition of the JAK2/STAT3 activation in macrophages, whereas OSM stimulation can activate the signaling pathway.

Atherosclerosis is a chronic inflammatory disease and its associated cardiovascular diseases constitute the leading causes of morbidity and mortality in developed countries (1, 2). Accumulated evidence demonstrates that atherosclerosis is initiated by endothelial activation and dysfunction, which is caused by disrupted shear stress and results in increased permeability for LDLs in the arterial wall (3). In the progression of atherosclerosis, the secretion of adhesion molecules by the activated endothelium leads to the adhesion and recruitment of monocytes into the subendothelium, where they differentiate into macrophages and subsequently phagocytize modified LDL to form lipid-laden foam cells (4). Foam cells exacerbate the inflammatory response and concomitantly promote the recruitment of monocytes and other inflammatory cells, causing the death of lesion-resident endothelial cells, macrophages, and smooth muscle cells (SMCs) and contributing to the instability of atherosclerotic plaques (5, 6). The rupture of unstable atherosclerotic plaques, which releases thrombogenic material into the blood stream, causes platelet activation and blood clotting and leads to myocardial infarction or stroke (6). Although the underlying mechanisms responsible for the progression of atherosclerotic plaques have been extensively studied, there is a big challenge for translating these discoveries into efficient treatments for atherosclerosis. At present, it is important for us to improve the understanding of the molecular regulation network for atherosclerosis and identify the potential targets for the development of effective preventive and therapeutic strategies.

Oncostatin M (OSM) is a member of the gp130 (or IL-6/LIFR) cytokine family and has an important effect on hematopoietic, immunologic, and inflammatory networks depending on the target cell by binding to the heterodimeric membrane receptor comprising the OSM-specific subunit [OSM receptor (OSMR)] and gp130 (7, 8). Before recruiting gp130, OSM engages a receptor, OSMR-β, to initiate the activation of several transcription factors, including signal transducer and activator of transcription (STAT)3 and MAPK, both of which are important modulators in the inflammatory response. Recently, epidemiological evidence has shown that an elevated OSM level in the serum is positively correlated with the severity of atherosclerosis (9). More importantly, OSM was found to exert critical effects on multiple cell types implicated in atherogenesis. First, OSM is produced by activated macrophages, monocytes, T cells, and dendritic cells (7) and can upregulate the expression of LDL receptor (10). Second, OSM has been reported to stimulate endothelial cells to secrete vascular cell adhesion molecule 1 (VCAM-1) and induce microvascular angiogenesis. Furthermore, it has been demonstrated that OSM can induce an inflammatory response in human aortic adventitial fibroblasts or SMCs (11). Notably, increased OSM and OSMR-β expression was found in the atherosclerotic vessel wall of mice (12). In addition, disruption of the OSMR gene in mice results in the development of mature onset obesity and systemic insulin resistance by regulating the function of macrophages (13, 14). This collective evidence strongly suggests a pro-atherosclerotic function of OSM/OSMR-β. However, the exact effect and underlying mechanism of OSM/OSMR-β on the development of atherosclerosis remains unclear.

Here, we found enhanced OSMR-β expression during atherosclerosis in humans and mice. More importantly, we discovered that OSMR-β deficiency attenuated the development of atherosclerosis and improved plaque stability partially through the inhibition of JAK2/STAT3 signaling.

## MATERIALS AND METHODS

### Animals and diet

All of the animal experiments were approved by the Animal Care and Use Committee of Renmin Hospital of Wuhan University. The OSMR-β KO mice that we utilized in our study were on C57BL/6J background. To purify the background, the female OSMR-β^−/−^ mice purchased from RIKEN BioResource Center (RBRC 02711) were first crossbred with male C57BL/6, and then the male F1 generation mice (OSMR-β heterozygous) were obtained and mated with female C57BL/6 mice for the F2 generation mice (OSMR-β heterozygous). The F2 generation mice were then repeatedly crossed with C57BL/6J mice until F9 generation (OSMR-β heterozygous). Finally, these F9 mice were then crossed to yield OSMR-β KO (pure C57BL/6J background) mice. Then, the OSMR-β^−/−^ mice (on a C57BL/6J background) were crossed with ApoE-deficient (ApoE^−/−^) mice to generate double KO mice. Eight-week-old male mice were used in this study and were maintained on a high-fat diet (HFD) or normal chow (NC) for up to 28 weeks. Body weights and sera were monitored or collected at the beginning of the experiment and when the animals were euthanized. The experimenters were blinded to the genotypes of the animals.

### Bone marrow transplantation

Donor bone marrow was isolated from male OSMR-β^−/−^ApoE^−/−^ or ApoE^−/−^ mice by flushing femurs and tibias with sterile PBS. Male ApoE^−/−^ recipient mice (8–10 weeks old) were irradiated with two doses of 5.5 Gy radiation 4 h apart, for a total of 11 Gy, prior to transplantation. For transplantation, each irradiated mouse was injected with 5 × 10^7^ donor bone marrow cells via retro-orbital venous plexus injection. Four weeks after transplantation, we extracted genomic DNA from the circulating blood leukocytes and genotyped the mice using PCR. Then, the mice were placed on a HFD for 16 weeks.

### Human specimens

Human studies were approved by the ethics committee of the Renmin Hospital of Wuhan University. Briefly, the atherosclerotic plaques were collected from the right coronary artery of patients with coronary heart disease (CHD) and heart failure who were undergoing heart transplantation. While the arteries as the control sample were obtained from normal donors who were unsuitable for transplantation without cardiovascular diseases. Written informed consent was obtained from the families of prospective heart donors.

### Quantitation and assessment of atherosclerotic plaques

Mice placed on a HFD were anesthetized with isoflurane followed by cervical dislocation. The entire aorta, including the subclavian and the right and left common carotid arteries, was collected from the base of the ascending aorta, whereas the heart and brachiocephalic arteries were harvested and fixed in 4% paraformaldehyde. Oil Red O staining was performed to analyze the en face atherosclerotic lesions of the aorta, as previously described (15). The heart and brachiocephalic arteries were dehydrated and embedded in paraffin or OCT and then sliced into 5 μm-thick sections for use in histological procedures. Then, the aortic roots and brachiocephalic arteries were stained with H&E for morphology, picrosirius red (PSR) to evaluate collagen deposition, and Oil Red O to detect lipid accumulation. Morphometric analysis was performed as described previously (15).

### Quantitative real-time PCR and Western blot analysis

Total RNA was extracted from whole aortas and cultured macrophages using TRIzol reagent (Invitrogen, Carlsbad, CA) and DNase-treated RNA was reverse transcribed with the use of a Transcriptor First-Strand cDNA synthesis kit (Roche, Indianapolis, IN). LightCycler 480 SYBR Green 1 Master Mix (Roche) and the LightCycler 480 QPCR system (Roche) were used to perform the quantitative real-time PCR analysis. The relative level of the mRNAs was calculated by normalization to the level of GAPDH mRNA. The whole aortas and cultured macrophages were lysed in lysis buffer, and 50 mg of extracted protein was separated on 8–12% SDS-PAGE gels and transferred to polyvinylidene difluoride membranes (Millipore, Bedford, MA). The membranes were probed with various primary antibodies overnight at 4°C. After incubation with secondary antibodies, the membranes were treated with ECL reagents (170-5061; Bio-Rad). Protein expression levels were quantified with Image Lab software version 5.1 (Bio-Rad) and normalized to the loading control, GAPDH. The information for the antibodies used in immunoblot analysis is shown in **Table 1**.

**TABLE 1.** The information for antibodies used in immunoblot

Antibody	Product Code	Origin	Molecular Mass (kDa)
OSMR-β	Santa Cruz (sc30011)	Rabbit	180
p-JAK2	CST (3776)	Rabbit	125
JAK2	CST (3230)	Rabbit	125
p-STAT3	CST (9145)	Rabbit	86
STAT3	CST (4904)	Rabbit	86
p-STAT1	CST (9167)	Rabbit	91
STAT1	CST (9172)	Rabbit	91
p-STAT5	Bioworld (BS4184)	Rabbit	90
STAT5	Bioworld (BS1995)	Rabbit	90
CD36	AbD (MCA2748)	Rat	88
ABCA-1	Abcam (ab18180)	Mouse	254
GAPDH	CST (2118)	Rabbit	37

### Immunofluorescence

The staining protocols used were described previously (15). Cross-sections of the aortic sinus were blocked in PBS containing 10% goat serum, incubated overnight at 4°C with primary antibodies, washed in PBS, and then incubated with the appropriate secondary antibody for 1 h. Secondary antibodies included goat anti-chicken IgY (H&L, DyLight® 488, ab96947; Abcam), Alexa Fluor 488-conjugated goat anti-mouse IgG (A11001; Invitrogen), and Alexa Fluor 568-conjugated anti-rabbit IgG (A11011, Invitrogen). The nuclei were labeled with DAPI. Images were acquired using a fluorescence microscope (Olympus, Tokyo, Japan) with DP2-BSW software (version 2.2) and were analyzed with Image Pro Plus 6.0. The information for the primary antibodies used in immunofluorescence analysis is shown in **Table 2**.

**TABLE 2.** Antibodies for Immunofluorescence staining

Antibody	Concentration	Sources of Species	Catalog Number
Anti-Mac3	1:50	Rat	550292
Anti-CD68	1:100	Rat	MCA1957
Anti-SMA	1:100	Mouse	ab7817
Anti-p-STAT3	1:100	Rabbit	9145
Anti-OSMR-β	1:200	Rabbit	Sc30011

### Cell culture and studies of macrophage lipid accumulation

Bone marrow-derived macrophages (BMDMs) isolated from both femurs and tibias of ApoE^−/−^ mice were harvested under sterile conditions. Approximately 5 × 10^7^ nucleated bone marrow cells were obtained from each mouse, and then cells were cultured in 10 ml RPMI containing 10% fetal bovine serum and MCSF (50 ng/ml). For in vitro studies, primary peritoneal macrophages pretreated with thioglycolate were isolated from OSMR-β^−/−^ApoE^−/−^ and ApoE^−/−^ mice. After serum starvation for 24 h, the macrophages were incubated with 15 μg/ml Ox-LDL for 24 h. Finally, the cells were fixed with paraformaldehyde and stained with Oil Red O to detect the lipid accumulation capacity of macrophages.

### Statistical analysis

All data are expressed as the mean ± SE. Differences among groups were analyzed with a two-tailed Student’s *t*-test using Statistical Package for the Social Sciences software, version 18.0. *P* < 0.05 was considered statistically significant.

## RESULTS

### OSMR-β accumulates in human and mouse atherosclerotic lesions

**Fig. 1.**
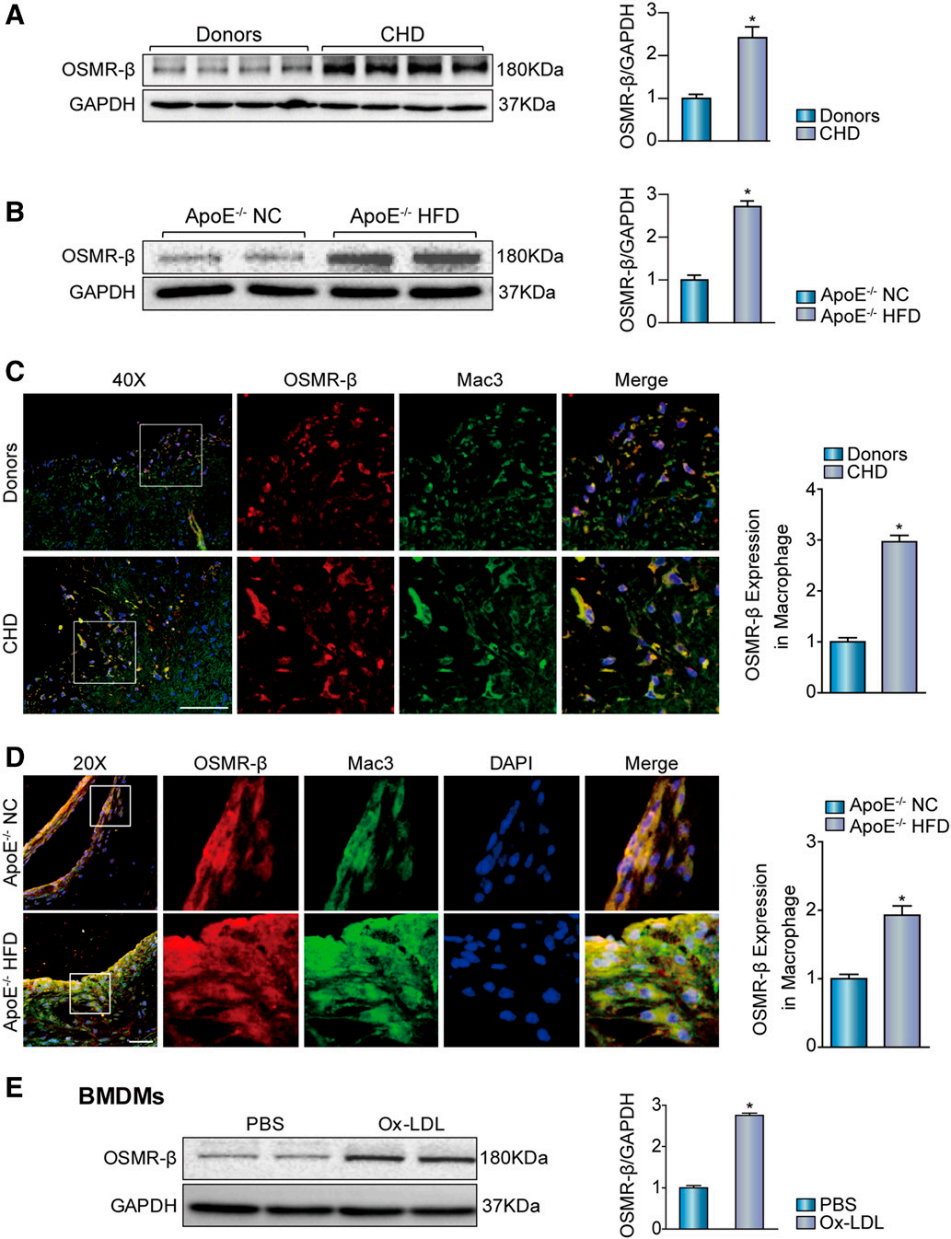
Enhanced OSMR-β expression in the atherosclerotic plaques of ApoE^−/−^ mice and patients with CHD. A: The expression of OSMR-β in the coronary arteries of normal donors and patients with CHD (n = 4, *P < 0.05 versus donor). B: The expression of OSMR-β in aortas from ApoE^−/−^ mice fed NC or a HFD (n = 4, *P < 0.05 versus control). C, D: Double immunofluorescence staining for OSMR-β (red) and Mac3 (macrophage, green) in the coronary arteries of normal donors and patients with CHD and in the aortic sinuses from ApoE^−/−^ mice fed NC or a HFD (scale bar = 50 μm). The quantification was carried out by normalizing the fluorescence intensity of the OSMR-β-positive area with the Mac3-positive area in the atherosclerotic plaque. E: OSMR-β expression in BMDMs upon 15 μg/ml Ox-LDL stimulation for 24 h. *P < 0.05 versus control group.

To determine whether OSMR-β is involved in the pathologic processes of atherosclerosis, we first investigated the expression level of OSMR-β in atherosclerotic lesions in human right coronary artery tissue from patients with CHD and arteries without plaque from normal heart donors who failed to qualify for heart transplantation for noncardiac reasons. Western blot analysis showed that the OSMR-β expression level in the coronary arteries from CHD patients was dramatically increased relative to that in donor tissue (**Fig. 1A**). Subsequently, to explore the OSMR-β expression pattern in atherosclerotic lesions harvested from the mouse model, we grouped atherosclerosis-prone ApoE^−/−^ mice by feeding them either NC or a HFD for 28 weeks, and the result indicated that HFD feeding significantly induced OSMR-β expression in the mouse aortas (Fig. 1B). In addition, double-immunofluorescence staining with OSMR-β and Mac3 (a marker of macrophages) revealed an increased OSMR-β expression, which located mainly in the macrophages of the atherosclerotic lesions of patients with CHD and the HFD-induced ApoE^−/−^ mouse model (Fig. 1C, D). Additionally, we also observed a significant increase of OSMR-β expression in the BMDMs upon Ox-LDL stimulation (Fig. 1E). Collectively, these data showed the upregulation of OSMR-β mainly in macrophages in atherosclerotic lesions, indicating its potential biological significance in both mice and humans.

### OSMR-β deficiency attenuates the development of atherosclerosis

**Fig. 2.**
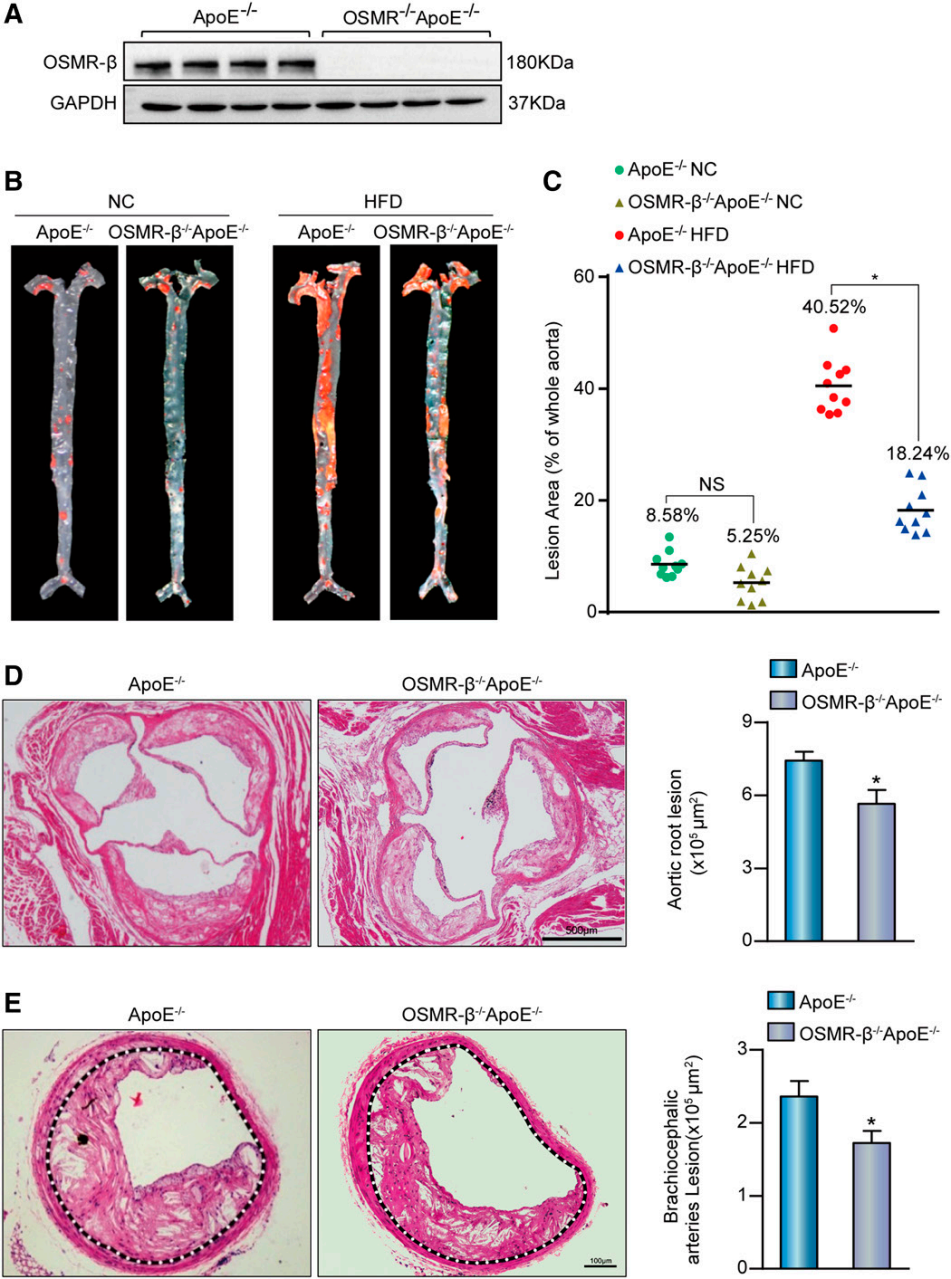
Deletion of OSMR-β ameliorated the development of atherosclerosis. A: Genotyping of OSMR-β^−/−^ApoE^−/−^ mice and ApoE^−/−^ littermates. B, C: En face analysis of aortas from OSMR-β^−/−^ApoE^−/−^ mice and ApoE^−/−^ littermates fed NC or a HFD; aortas were stained with Oil Red O (n = 10). D, E: Left panel, representative images of the aortic sinus or brachiocephalic arteries from OSMR-β^−/−^ApoE^−/−^ and ApoE^−/−^ mice stained with H&E. Right panel, quantification of the atherosclerotic lesion area (n = 6). *P < 0.05 compared with ApoE^−/−^.

Considering that OSMR-β expression was significantly increased in the aortas of ApoE^−/−^ mice fed a HFD, we proposed that endogenous OSMR-β plays a critical role in the development of atherosclerosis. To test this hypothesis, OSMR-β^−/−^ApoE^−/−^ mice were generated by crossing the OSMR-β^−/−^ strain with ApoE^−/−^ mice, and the whole aortas of mice were collected and identified by Western blot analysis (**Fig. 2A**). Then, male OSMR-β^−/−^ApoE^−/−^ mice and ApoE^−/−^ littermates were subjected to a HFD for 28 weeks, starting from 8 weeks of age. It is well-recognized that a HFD leads to hyperlipidemia, and large clinical studies have provided strong support for the association between plasma lipid levels and the risk of cardiovascular events (16). We measured the body weight and the triglyceride, total cholesterol, VLDL, LDL, IDL, and HDL levels in the serum and found no differences between ApoE^−/−^ mice and OSMR-β^−/−^ApoE^−/−^ mice (**Table 3**). Through en face analysis of atherosclerotic lesions by Oil Red O staining of the entire aorta, we noticed that a limited number of lesions developed in the proximal aortas of mice treated with NC, and a reduction in the total plaque area in OSMR-β^−/−^ApoE^−/−^ mice relative to ApoE^−/−^ mice, whereas this difference was not statistically significant. In contrast, the total plaque area in OSMR-β^−/−^ApoE^−/−^ mice was almost 55% lower than that in ApoE^−/−^ mice fed a HFD (Fig. 2B, C). In addition, H&E staining was performed to analyze the advanced atherosclerotic lesions in cross-sections of the aortic sinus and brachiocephalic arteries. In accordance with the results of the en face analysis, the area of lesions in the aortic root and brachiocephalic arteries was reduced by 24% and 26%, respectively, in OSMR-β^−/−^ApoE^−/−^ mice (Fig. 2D, E). Taken together, these results demonstrated that OSMR ablation in ApoE^−/−^ mice attenuated HFD-induced atherosclerosis and that this effect was not dependent on dyslipidemia.

**TABLE 3.** Metabolic characteristics of OSMR-β^−/−^ApoE^−/−^ mice and ApoE^−/−^ mice

Index	Timing	ApoE^−/−^	OSMR-β^−/−^ApoE^−/−^	*P*
Body weight (g)	Before HFD	23.89 ± 0.37	25.57 ± 0.83	0.075
	28 week HFD	35.03 ± 1.26	34.60 ± 1.39	0.819
TG (mg/dl)	28 week HFD	81.17 ± 0.48	83.56 ± 0.80	0.684
TC (mg/dl)	28 week HFD	513.88 ± 6.20	510.49 ± 6.72	0.715
VLDL (mg/dl)	28 week HFD	542.66 ± 5.86	553.12 ± 6.50	0.247
LDL (mg/dl)	28 week HFD	346.32 ± 3.32	355.39 ± 3.69	0.085
IDL (mg/dl)	28 week HFD	210.5 ± 2.89	216.38 ± 6.38	0.417
HDL (mg/dl)	28 week HFD	50.16 ± 0.98	48.33 ± 0.23	0.099

TG, triglyceride; TC, total cholesterol.

### OSMR-β deficiency decreases the necrotic area and enhances the stability of plaques

**Fig. 3.**
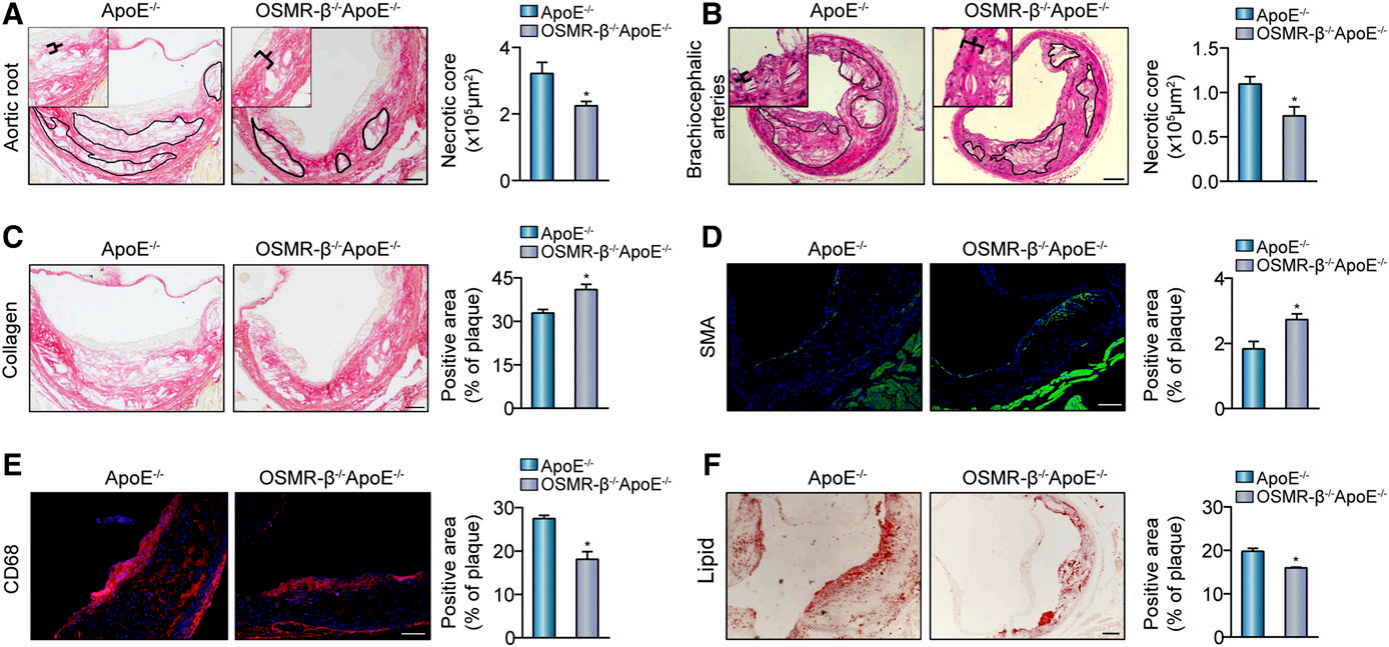
OSMR-β ablation reduces the area of necrotic core and enhances plaque stability. A, B: Left panel, representative images used to analyze the necrotic area in aortic roots and brachiocephalic arteries from OSMR-β^−/−^ApoE^−/−^ and ApoE^−/−^ mice. Right panel, quantitation of the necrotic area (n = 6). C–E: Histological analysis of plaque stability in the aortic sinus. C: PSR staining for the detection of collagen content. D: Immunofluorescence analysis of SMA for the detection of SMC content. E: Immunofluorescence analysis of CD68 for the detection of macrophage infiltration. F: Oil Red O staining for the detection of lipid accumulation. *P < 0.05 versus ApoE^−/−^.

Current evidence suggests that the physical disruption of plaques may trigger thrombosis and thus promote the sudden expansion of atheromatous lesions, with the level of collagen being crucial for the stability of plaques. We conducted a morphological analysis to evaluate the necrotic core via PSR staining in the aortic root and H&E staining in the brachiocephalic arteries. Analysis of the lesions indicated by the black markers showed that the total necrotic core area was significantly smaller in OSMR-β^−/−^ApoE^−/−^ mice than in ApoE^−/−^ mice (**Fig. 3A, B**). Additionally, other properties of plaque composition contributing to plaque instability were investigated, including collagen and SMC content covering the fibrous cap, macrophage numbers, and lipid accumulation. First, as shown in Fig. 3C, the percentage of collagen in the plaques was more abundant in OSMR-β^−/−^ApoE^−/−^ mice than ApoE^−/−^ littermates (Fig. 3C). Second, immunofluorescence staining showed that the smooth muscle actin (SMA)-positive area in the plaques was significantly increased by 49% in OSMR-β^−/−^ApoE^−/−^ mice, whereas the CD68-positive area was obviously decreased by almost 34% in OSMR-β^−/−^ApoE^−/−^ mice relative to ApoE^−/−^ mice (Fig. 3D, E). Finally, Oil Red O staining of the lipid area revealed a 19% reduction in lipid accumulation in the lesions from OSMR-β^−/−^ApoE^−/−^ mice relative to those from ApoE^−/−^ controls (Fig. 3F). Taken together, these data illustrated that OSMR-β deficiency on an ApoE^−/−^ background plays a protective role in the progression of atherosclerotic lesions via enhancing the stability of atherosclerotic plaques.

### OSMR-β deficiency decreases inflammation in atherogenesis

**Fig. 4.**
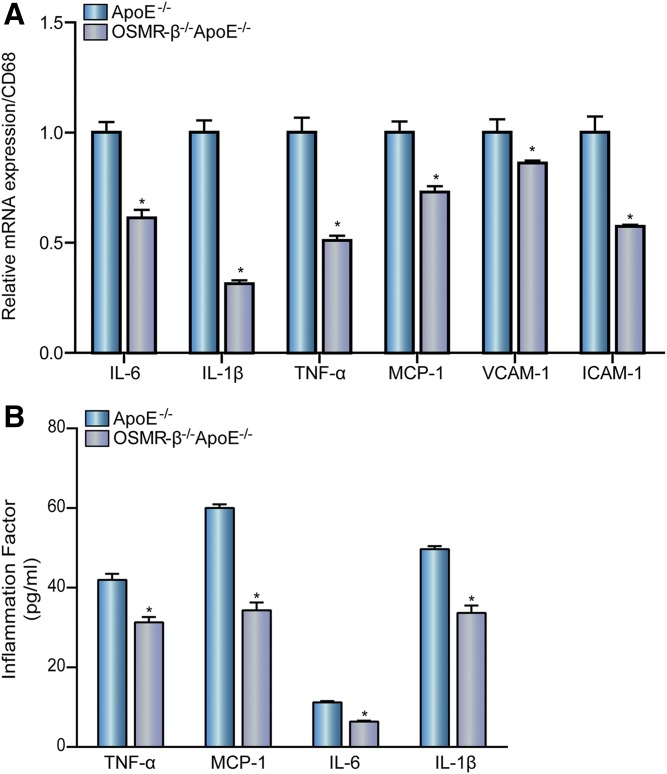
OSMR-β deficiency decreases atherosclerosis-induced inflammation. A: Real-time PCR analysis of pro-inflammatory cytokine expression in the whole aortas of OSMR-β^−/−^ApoE^−/−^ and ApoE^−/−^ mice compared with CD68 expression (n = 3). B: Detection of MCP-1, TNF-α, IL-6, and IL-1β expression in the serum with ELISA (n = 6). *P < 0.05 compared with ApoE^−/−^.

Atherosclerosis is characterized as an inflammatory process, with inflammation playing an important role in all stages of atherosclerosis from initiation through progression and ultimately the occurrence of thrombotic complications (17). Real-time PCR analysis showed that the levels of multiple pro-inflammatory genes, such as IL-6, IL-1β, TNF-α, monocyte chemotactic protein 1 (MCP-1), intercellular cell adhesion molecule-1 (ICAM-1), and VCAM-1, were significantly decreased compared with CD68 expression in the atherosclerotic lesions of OSMR-β^−/−^ApoE^−/−^ mice relative to ApoE^−/−^ mice (**Fig. 4A**). The level of inflammatory factors in the serum was also measured using ELISA. As expected, the levels of TNF-α, MCP-1, IL-6, and IL-1β secreted into the sera of OSMR-β^−/−^ApoE^−/−^ mice were significantly lower than those in control mice (Fig. 4B). These results demonstrate that OSMR-β deficiency decreases inflammation in the pathogenesis of atherosclerosis.

### OSMR-β activates the JAK2/STAT3 signal transduction pathway in atherosclerosis in vivo and in vitro

**Fig. 5.**
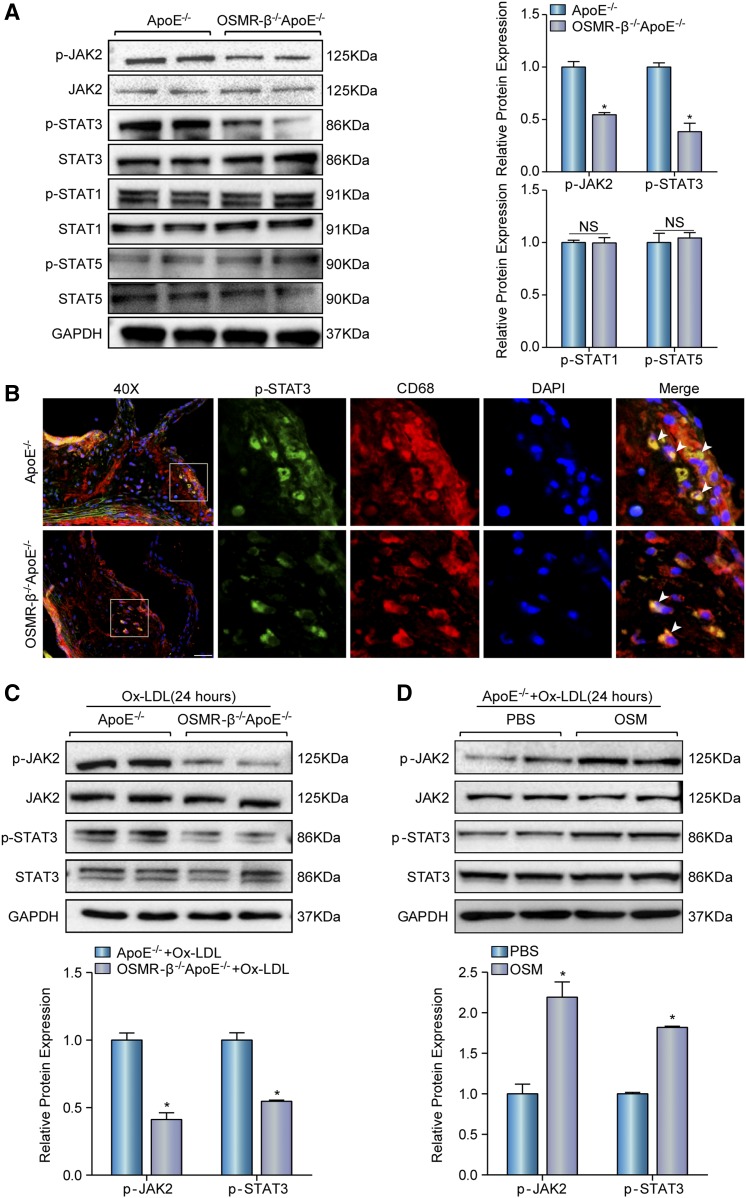
OSMR-β deficiency inhibits the activation of JAK2/STAT3 signaling. A: Western blot analysis of the expression of phosphorylated and total JAK2, STAT3, STAT1, and STAT5 in the whole aortas of OSMR-β^−/−^ApoE^−/−^ and ApoE^−/−^ littermates. Quantitation of relative phosphorylated protein expression after normalization to each total protein expression, respectively (n = 3). B: Immunofluorescence costaining of phosphorylated STAT3 (green) and CD68 (red) in atherosclerotic plaques (scale bar = 50 μm). C: Western blot analysis of phosphorylated and total JAK2 and STAT3 in peritoneal macrophages from OSMR-β^−/−^ApoE^−/−^ and ApoE^−/−^ littermates upon 15 μg/ml Ox-LDL treatment for 24 h. The results present phosphorylated protein expression compared with the total protein expression, respectively (n = 3). D: The JAK2 and STAT3 expression in macrophages from ApoE^−/−^ mice upon PBS or OSM treatment. *P < 0.05 compared with control group.

Several signaling pathways, including the JAK/STAT and MAPK pathways, are stimulated by gp130 cytokines (7). To identify a potential molecular mechanism by which OSMR-β accelerated the development of atherosclerosis, we examined the activation state of the JAK-STAT signaling pathway. Western blotting showed that the activation of the JAK2/STAT3 cascade was significantly inhibited by OSMR-β ablation, whereas the level of total and phosphorylated STAT1 and STAT5 exhibited no statistical significance (**Fig. 5A**). Furthermore, costaining of p-STAT3 and CD68 showed that p-STAT3 was predominantly located in macrophages in both groups and was markedly reduced in OSMR-β^−/−^ApoE^−/−^ mice (Fig. 5B). Concomitantly, we also observed a similar pattern in the JAK2/STAT3 cascade in peritoneal macrophages treated with Ox-LDL that were isolated from OSMR-β^−/−^ApoE^−/−^ mice and ApoE^−/−^ mice (Fig. 5C). Additionally, we found the activity of phosphorylated JAK2/STAT3 indeed increased in the peritoneal macrophages from ApoE^−/−^ mice treated with OSM under Ox-LDL stimulation (Fig. 5D). Taken together, these data suggest that the loss of OSMR-β attenuates the development of atherosclerosis, at least partly, via inhibiting the JAK2/STAT3 signal transduction pathway in macrophages.

### OSMR-β ablation in bone marrow-derived cells attenuates atherogenesis

**Fig. 6.**
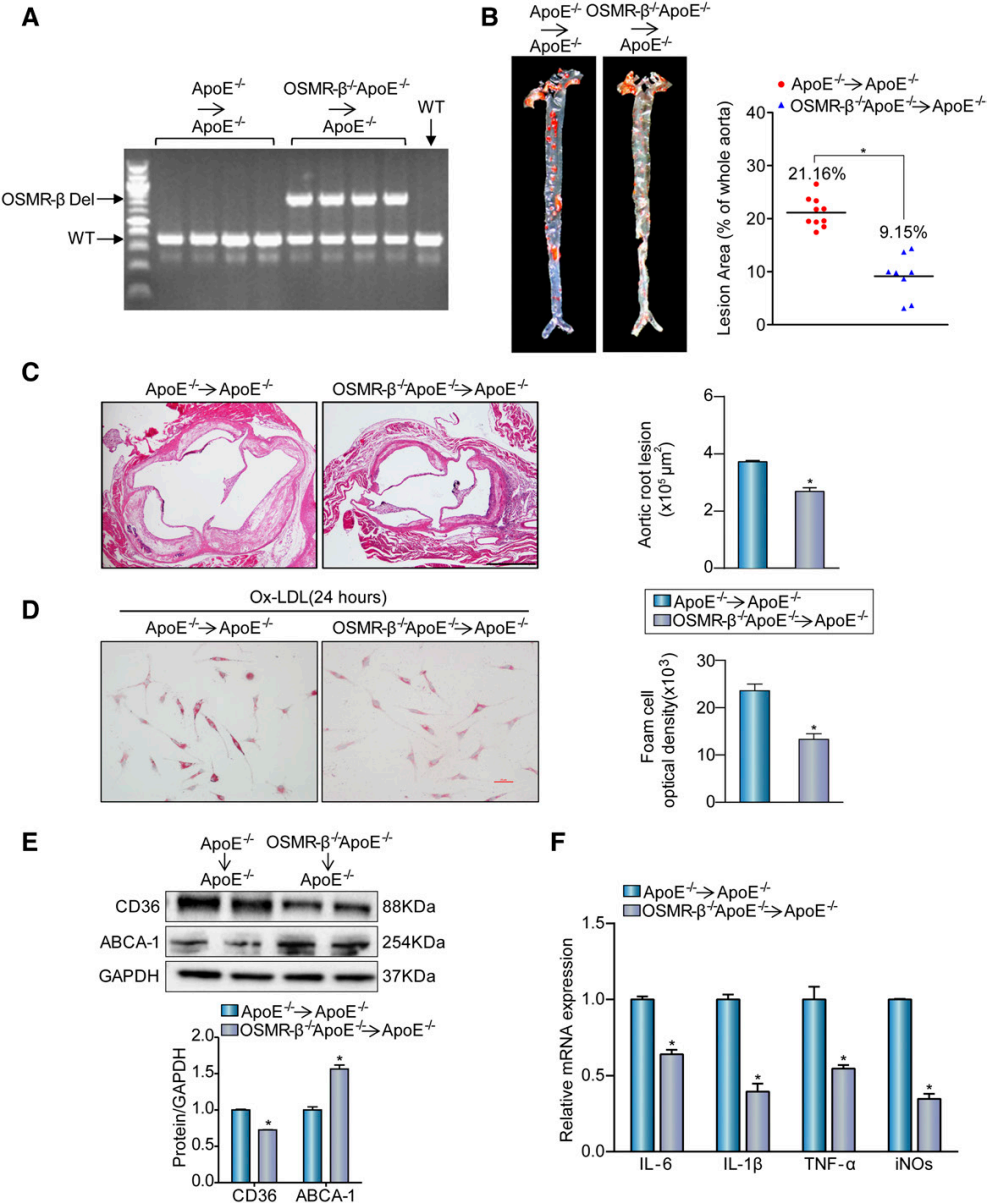
The absence of OSMR-β from bone marrow-derived cells attenuates atherogenesis. A: DNA was isolated from whole blood for genotyping to confirm genome reorganization with PCR. B: Left panel, Oil Red O staining for en face analysis of atherosclerotic plaques in aortas from OSMR-β^−/−^ApoE^−/−^→ApoE^−/−^ (n = 8) and ApoE^−/−^→ApoE^−/−^ (n = 10) mice. Right panel, analysis of the atherosclerotic lesion area (n = 10 or 8, each group). C: Left panel, H&E staining of the aortic root from OSMR-β^−/−^ApoE^−/−^→ApoE^−/−^ mice and ApoE^−/−^→ApoE^−/−^ mice. Right panel, quantification of the atherosclerotic lesion area (scale bar = 500 μm, n = 6). D: Oil Red O staining of peritoneal macrophages from OSMR-β^−/−^ApoE^−/−^→ApoE^−/−^ mice and ApoE^−/−^→ApoE^−/−^ mice treated with Ox-LDL. E: Western blot analysis of CD36 and ABCA-1 expression in OSMR-β^−/−^ApoE^−/−^→ApoE^−/−^ and ApoE^−/−^→ApoE^−/−^ mice. F: Real-time PCR analysis of pro-inflammatory cytokine expression in macrophages from OSMR-β^−/−^ApoE^−/−^→ApoE^−/−^ mice and ApoE^−/−^→ApoE^−/−^ mice treated with Ox-LDL (n = 3). *P < 0.05 compared with ApoE^−/−^→ApoE^−/−^ group.

Bone marrow transplantation (BMT) is a common approach to reconstitute the immune systems of mice that have been subjected to marrow-ablative doses of radiation and can be used to assess the contribution of hematopoietic cells of a specific genotype to disease pathogenesis (18). Bone marrow-derived cells from OSMR-β^−/−^ApoE^−/−^ or ApoE^−/−^ mice were transferred to ApoE^−/−^ recipients to examine the impact of macrophage OSMR-β deficiency on atherosclerotic lesions. Bone marrow reconstitution was confirmed 4 weeks after BMT by PCR of genomic DNA from the peripheral blood (**Fig. 6A**). At this time, mice were fed a HFD for 16 weeks. Then, their aortas were stained with Oil Red O. The surface area of the atherosclerotic lesions in the aortas of the OSMR-β^−/−^ApoE^−/−^→ApoE^−/−^ group was remarkably smaller than those in the ApoE^−/−^→ApoE^−/−^ mice (Fig. 6B). Moreover, morphometric analysis of aortic root lesions showed a reduction in the total lesion area in the aortic sinus from the OSMR-β^−/−^ApoE^−/−^→ApoE^−/−^ mice compared with the ApoE^−/−^→ApoE^−/−^ group (Fig. 6C). The uptake of lipoprotein by macrophages facilitates foam cell formation, and then foam cells exacerbate the inflammatory response and play an important role in atherosclerosis (3, 4). To address whether macrophage OSMR-β deficiency mediates lipid accumulation and pro-inflammatory cytokine expression upon stimulation, peritoneal macrophages pretreated with thioglycolate were isolated from OSMR-β^−/−^ApoE^−/−^→ApoE^−/−^ and ApoE^−/−^→ApoE^−/−^ mice and treated with 15 μg/ml Ox-LDL for 24 h. As shown in Fig. 6D, the OSMR-β^−/−^ApoE^−/−^→ApoE^−/−^ group exhibited a clear and consistent reduction in Oil Red O staining and a decreased expression of CD36 was observed in OSMR-β deficiency in macrophages, whereas ABCA-1 showed an increased expression (Fig. 6E). Additionally, the mRNA levels of pro-inflammatory cytokines were also decreased in macrophages from OSMR-β^−/−^ApoE^−/−^→ApoE^−/−^ mice (Fig. 6F). Together, these results demonstrate that OSMR-β deficiency in macrophages attenuated the development of atherosclerosis.

## DISCUSSION

The following principal observations made by the current study uncovered the central role of macrophage-derived OSMR-β in the regulation of atherogenesis. First, OSMR-β expression was induced in atherosclerotic lesions and was mainly located in macrophages. Second, OSMR-β deficiency protected mice against HFD-induced atherosclerosis, and BMT demonstrated that the absence of OSMR-β from bone marrow-derived cells improved atherosclerosis. Third, OSMR-β deficiency increased the stability of atherosclerotic plaques, decreased the inflammatory response, and inhibited the formation of foam cells. Mechanistically, the role of OSMR-β in the development of atherosclerosis is partially mediated through regulation of the JAK2/STAT3 signaling pathway.

Decades of research have demonstrated that persistent chronic inflammation accelerates the progression of atherosclerotic lesions in the artery wall throughout the different stages of the disease (19–22). OSM is a secreted cytokine produced by activated macrophages, monocytes, T cells, and dendritic cells (7). Through engagement of OSMR-β, this cytokine can regulate the expression of IL-6 (23), G-CSF, and GM-CSF (24). Multiple studies have shown that an elevated OSM level in the serum is associated with the coronary stenosis score and the extent of coronary atherosclerosis (24). Consistently, in the current study, we found that OSMR-β expression was induced in the atheroma of humans and ApoE^−/−^ mice, as well as in the isolated BMDMs upon Ox-LDL stimulation. However, whether increased OSMR-β is part of a compensatory mechanism or a key regulatory factor in the development of atherosclerosis requires further elucidation. Recently, our group reported that OSMR-β alleviates obesity-induced hepatic insulin resistance and steatosis (25), which are risk factors for the development of atherosclerosis. In the present study, OSMR-β^−/−^ApoE^−/−^ mice and their control littermates were fed a HFD for 28 weeks; we did not observe a significant difference in the serum lipid profiles of OSMR-β-null and OSMR-β-expressing mice on an ApoE^−/−^ background. However, we found that OSMR-β^−/−^ApoE^−/−^ mice developed smaller lesions than their control littermates in three different locations: the aortic root, the brachiocephalic artery, and the en face aorta. These results suggest a causal link between OSMR-β and atherosclerosis rather than a compensatory response, and this effect was independent of the change in lipid metabolism. Meanwhile, the BMT further demonstrated that the inhibition of atherogenesis was dependent on the effect of OSMR-β deficiency in macrophages. Although the ablation of OSM signaling can affect the red blood cell and platelet numbers (26, 27), we did not observe a significant difference between ApoE^−/−^ and OSMR-β^−/−^ApoE^−/−^ mice in this regard here.

As the most prominent inflammatory cells in the plaque, activated macrophages are fundamental contributors (28) to plaques and can produce diverse pro-inflammatory cytokines, such as TNF-α, IL-1β, IL-6, and IL-12, as well as anti-inflammatory cytokines, such as IL-10 and TGF-β, which have an anti-atherogenic effect (29, 30). In our work, we noticed that OSMR-β was mainly expressed in macrophages. Additionally, we found that OSMR-β^−/−^ApoE^−/−^ chimeras showed a reduction in atherosclerotic plaques in the whole aorta and aortic sinus, and that OSMR-β deletion from macrophages decreased foam cell formation upon stimulation with Ox-LDL, which further confirmed that the absence of OSMR-β in macrophages was responsible for the atheroprotective effect.

Furthermore, analysis of the plaques in the aortic sinus demonstrated that OSMR-β^−/−^ApoE^−/−^ mice exhibited smaller numbers of invading macrophages. In addition, a key observation of this study was that OSMR-β^−/−^ApoE^−/−^ mice exhibited reduced induction of vascular inflammation, characterized by the decreased expression of pro-inflammatory cytokines, such as IL-6, IL-1β, and TNF-α, at the mRNA and serum protein levels. Interestingly, we also observed that OSMR-β^−/−^ApoE^−/−^ mice showed alleviated levels of MCP-1, VCAM-1, and ICAM-1, which mediate the adhesion and recruitment of monocytes into the arterial wall, where they differentiate into macrophages (3). Therefore, we demonstrated that OSMR-β deficiency in macrophages reduced the severity of atherosclerosis by decreasing inflammation.

Clinically, the occurrence of most cardiovascular events is largely attributed to the progression and rupture of unstable atheromatous plaques accompanied by superimposed thrombosis, which will result in partial or complete occlusion of the vessel lumen and downstream ischemia, such as in myocardial infarction and stroke. Plaque morphology and the size of the necrotic core, rather than the plaque size, are crucial for plaque stability (31). In our work, we also tested to determine whether OSMR-β affects apoptosis and the results showed there was no significant difference between the two groups. Additionally, there are also some aspects of atherosclerotic plaques that contribute to the stability of the lesion, including the thinning of the fibrous cap, the infiltration of inflammatory cells, the abundant accumulation of lipids, and the proliferation and migration of SMCs (32, 33). In the present study, we observed that the total necrotic areas were reduced by 30% or 33%, respectively, in the aortic root and brachiocephalic arteries of OSMR-β^−/−^ApoE^−/−^ mice relative to ApoE^−/−^ mice. Additionally, OSMR-β^−/−^ApoE^−/−^ mice exhibited a dramatic increase in the content of collagen and SMCs, but a decrease in macrophage infiltration and lipid accumulation. It has been demonstrated that the inflammation induced by TNF-α or IL-1β can promote matrix-degrading metalloproteinase expression and promote tissue remodeling (34, 35), and TNF-α facilitates oxidative stress in SMCs and accelerates vascular SMC apoptosis (36, 37). Correspondingly, previous studies have demonstrated that advanced lesions are characterized by the apoptosis and necrosis of macrophages or other cell types, which results in the release of lipids and inflammatory mediators and the formation of the necrotic core. These results suggest an important function of OSMR-β deficiency in the progression of atherosclerosis, particularly in maintaining plaque stability.

With these data, the present study provides cumulative evidence that OSMR-β has an important effect on the development of atherosclerosis, but the potential mechanism through which OSMR-β deficiency confers an atheroprotective effect remains unclear. It is known that the gp130-ligand interaction activates the JAK/STAT, MEK, and PI3K-Akt pathways (7). Previous work from our laboratory showed that OSMR-β could regulate obesity-induced metabolic disorders of hepatic and cerebral ischemia/reperfusion injury in a STAT3-dependent manner (38). In addition, the JAK/STAT pathway is involved in blood cell formation and immunity, macrophage activation and polarization (39). Furthermore, STAT3 phosphorylation is markedly increased in atherosclerotic lesions, and interfering with the STAT3 pathway in vivo prevents atherosclerotic lesion formation (40, 41). Therefore, these findings suggest that the JAK/STAT signaling pathway may be associated with the effects of OSMR-β on atherosclerosis. To address this hypothesis, we determined the state of JAK/STAT signaling in atherosclerotic lesions in OSMR-β-deficient mice. As expected, in vivo and in vitro data indicated that the phosphorylation of JAK2/STAT3 was downregulated in OSMR-β^−/−^ApoE^−/−^ mice relative to ApoE^−/−^ mice and OSM stimulation can activate the signaling pathway, whereas the phosphorylation of STAT1 and STAT5 was unchanged. Additionally, the expression of downstream molecules of STAT3 activation, such as ICAM-1 and VCAM-1 (42, 43), was decreased. Importantly, phosphorylated STAT3 mainly colocalized with the macrophage marker, CD68. These results suggested that altered JAK/STAT3 signaling was responsible for the anti-atherosclerotic effects of OSMR deficiency. In addition, previous studies have demonstrated that STAT3 and nuclear transcription factor-κB (NF-κB) interact at multiple levels and that STAT3 may account for the constitutive activation of NF-κB during chronic inflammation; this activation contributes to the transcriptional regulation of inflammatory cytokines, such as ICAM-1, VCAM-1, MCP-1, and IL-6, as well as matrix metalloproteinases (44, 45). Our results showed decreased expression of ICAM-1, VCAM-1, MCP-1, and IL-6, which can also be transcriptionally regulated by NF-κB (46), in OSMR-β^−/−^ApoE^−/−^ mice. Thus, we speculate that the potential mechanism underlined might be activation of the JAK2/STAT3 pathway mediated by OSMR-β in macrophages. Then, phosphorylated STAT3 forms a dimer and translocates into the nucleus to activate the transcription of genes containing STAT3 response elements, leading to the upregulation of several cytokines in monocytes and promoting the formation of atherosclerotic lesions.

Although important discoveries were made by these experiments, it should be noted that the important role of OSMR-β in atherosclerosis indicated by our study mainly revealed its effect in macrophages because we observed that OSMR-β was predominantly located in macrophages in atherosclerotic plaques and the BMT experiment further demonstrated that OSMR-β deficiency from macrophage was responsible for inhibition of atherogenesis. However, previous studies have demonstrated that OSM can stimulate endothelial cells to secrete VCAM-1, which contributes to microvascular endothelial cell angiogenesis (47) and the proliferation of rabbit aortic SMCs (48), as well as the induction of VEGF, IL-6, and COX-2 (49, 50). Indeed, many of these ideas are relevant and require further investigation.

In summary, our findings suggested that OSMR-β deficiency in macrophages alleviated HFD-induced atherogenesis and plaque instability, which were accompanied by decreased macrophage infiltration, suppressed vascular inflammation, and decreased foam cell formation. In addition, the anti-atherogenesis effect was partially attributed to the inhibition of the JAK2/STAT3 pathways. In this context, preventing the activation of the OSMR-β signaling pathway in macrophages could represent a new strategy for attenuating atherosclerosis.
